# Scope and efficacy of the broad-spectrum topical antiseptic choline geranate

**DOI:** 10.1371/journal.pone.0222211

**Published:** 2019-09-17

**Authors:** Joshua R. Greene, Kahla L. Merrett, Alexanndra J. Heyert, Lucas F. Simmons, Camille M. Migliori, Kristen C. Vogt, Rebeca S. Castro, Paul D. Phillips, Joseph L. Baker, Gerrick E. Lindberg, David T. Fox, Rico E. Del Sesto, Andrew T. Koppisch

**Affiliations:** 1 Department of Chemistry, Northern Arizona University, Flagstaff, AZ, United States of America; 2 Department of Chemistry, Dixie State University, St. George, UT, United States of America; 3 Department of Chemistry, The College of New Jersey, Ewing, NJ, United States of America; 4 Center for Materials Interfaces in Research and Application, Northern Arizona University, Flagstaff, AZ, United States of America; 5 Chemistry Division, Los Alamos National Laboratory, Los Alamos, NM, United States of America; Institut Pasteur, FRANCE

## Abstract

Choline geranate (also described as Choline And GEranic acid, or CAGE) has been developed as a novel biocompatible antiseptic material capable of penetrating skin and aiding the transdermal delivery of co-administered antibiotics. The antibacterial properties of CAGE were analyzed against 24 and 72 hour old biofilms of 11 clinically isolated ESKAPE pathogens (defined as *Enterococcus faecium*, *Staphylococcus aureus*, *Klebsiella pneumoniae*, *Acinetobacter baumanii*, *Pseudomonas aeruginosa*, and *Enterobacter sp*, respectively), including multidrug resistant (MDR) isolates. CAGE was observed to eradicate *in vitro* biofilms at concentrations as low as 3.56 mM (0.156% v:v) in as little as 2 hours, which represents both an improved potency and rate of biofilm eradication relative to that reported for most common standard-of-care topical antiseptics in current use. *In vitro* time-kill studies on 24 hour old *Staphylococcus aureus* biofilms indicate that CAGE exerts its antibacterial effect upon contact and a 0.1% v:v solution reduced biofilm viability by over three orders of magnitude (a 3log10 reduction) in 15 minutes. Furthermore, disruption of the protective layer of exopolymeric substances in mature biofilms of *Staphylococcus aureus* by CAGE (0.1% v:v) was observed in 120 minutes. Insight into the mechanism of action of CAGE was provided with molecular modeling studies alongside *in vitro* antibiofilm assays. The geranate ion and geranic acid components of CAGE are predicted to act in concert to integrate into bacterial membranes, affect membrane thinning and perturb membrane homeostasis. Taken together, our results show that CAGE demonstrates all properties required of an effective topical antiseptic and the data also provides insight into how its observed antibiofilm properties may manifest.

## Introduction

Antiseptics are one of the most important components of the arsenal of antibacterial materials used against nosocomial and community-acquired infections. In hospitals, antiseptics are invaluable both for their ability to disinfect surfaces and clean skin prior to medical procedures. Unfortunately, the number of materials recognized as antiseptic ingredients by the U.S. Food and Drug Administration (FDA) is very limited and in April 2019 the agency upheld its own 2016 ruling which determined that 28 of the 31 recognized ingredients to be no longer eligible for inclusion into consumer antiseptic products without a thorough clinical re-evaluation of their safety profiles.[1] Given the critical role these materials play in modern healthcare, the development of new antiseptics is now a priority.

A significant requirement of any effective antiseptic is a demonstrated ability to combat microbial biofilms. Microbial biofilms are widespread in clinical settings and responsible for the majority of chronic infections in patients worldwide.[2–5] Biofilms readily colonize biotic and abiotic surfaces alike, and are consequently associated with diseases that affect tissue (such as endocarditis, periodontitis, urinary tract infections and chronic/non-healing wounds) as well as corruption of indwelling medical devices. [6, 7] In addition to their role in the manifestation of infection, biofilms are inherently recalcitrant to antibiotic/antiseptic treatment due in part to a protective layer of exopolymeric substances (EPS) that covers the cells.[8, 9] The EPS layer is comprised of polysaccharides, nucleic acids, peptides and humic acids, and the presence of the layer serves to protect the underlying cells in a number of ways, including limiting diffusion of many small-molecule antibacterials.[8, 10, 11] The EPS layer is one of a number of factors that contributes to the observation that, microbial biofilms are 50–1000 times less sensitive to commonly prescribed antibiotics than their planktonic counterparts,[8] and biofilms of multidrug resistant organisms and metabolically inactive cells represent even more ominous hazards to patients.[12–14]

Our team and collaborators recently developed and characterized choline geranate (also described as Choline And GEranic acid, or CAGE) as a new antibiofilm agent/antiseptic.[15, 16] Ionic liquids (IL) and deep eutectic solvents (DES) are uniquely suited to combat microbial biofilms [17–20] due to the ability of IL/DES to disrupt hydrogen bonding networks found in recalcitrant biopolymers (such as the EPS) and to facilitate their dissolution.[21, 22] Although there has been some debate as whether CAGE is best classified as an IL or a DES,[23, 24] CAGE demonstrates a broad antibacterial efficacy and rapidly inactivates microbial pathogens *in vitro*. While these features are advantageous for a potential antiseptic, the low observed toxicity to mammalian cells and ability to facilitate transdermal delivery of pharmaceutical agents dissolved within the material illustrate the utility of CAGE within antimicrobial chemotherapy as well.[15, 16] The latter property has enabled co-treatment of subdermal infections with CAGE and clinical antibiotics within *in vitro* assays and murine models. [17,18] Taken together, these features highlight the significant potential CAGE may have as an antibacterial antiseptic to both decontaminate skin and to combat infections to skin and wounds. Despite the promise CAGE has shown, to date, little information of CAGE’s antibacterial activity on biofilm cultures of clinically-relevant pathogens exists, and no substantive mechanistic analysis of its antibacterial action has been pursued. In this work, we have pursued the first detailed examination of the effect of CAGE on sessile cultures of clinically relevant pathogens, including efforts to quantify its rate of eradication of mature biofilms and its interaction with the EPS. Computational modeling of CAGE’s interaction with a biological membrane complemented our antibiofilm assays to provide further insight into its potential molecular mechanism of action against select pathogens.

## Results

### Chemical Synthesis and Characterization of CAGE

**Fig 1:**
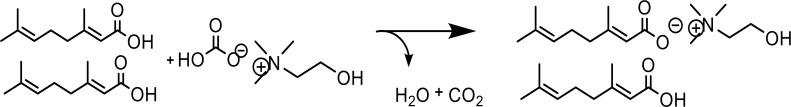
Synthesis of choline geranate. CAGE was synthesized with equimolar molar equivalents of cholinium cation, geranate anion, and protonated geranic acid from choline bicarbonate and geranic acid.

As reported previously, the synthesis of CAGE was conducted via salt metathesis using choline bicarbonate and geranic acid (Fig 1), followed by dehydration with a vacuum oven.

The reaction proceeded in quantitative yield, and compound purity was assessed via ^1^H and ^13^C NMR spectroscopy. NMR spectra, along with other physicochemical properties, were consistent with previous reports (S1 and S2 Figs). Conductivity of CAGE was found to be 43 mS/cm, which is similarly consistent with previous reports, however this value is observed to elevate with incomplete drying or as the hygroscopic material hydrates over time upon prolonged storage outside of a dessicator (S3 Fig). CAGE appears as a clear liquid with a faint yellow hue, but is observed to darken upon prolonged (>4 months) storage at room temperature. In our experience, hydrated samples of CAGE appear to undergo a change in color more rapidly and to a greater extent than those kept under rigorous anhydrous conditions, and as such conductance is a particularly useful means to infer hydration of the compound. In any case, we do not observe a marked diminishment of the antibacterial potency of CAGE as it ages, however all studies described here used preparations that were no more than 3 months old.

### Eradication of *S*. *aureus* biofilms occurs upon contact with neat CAGE

**Fig 2:**
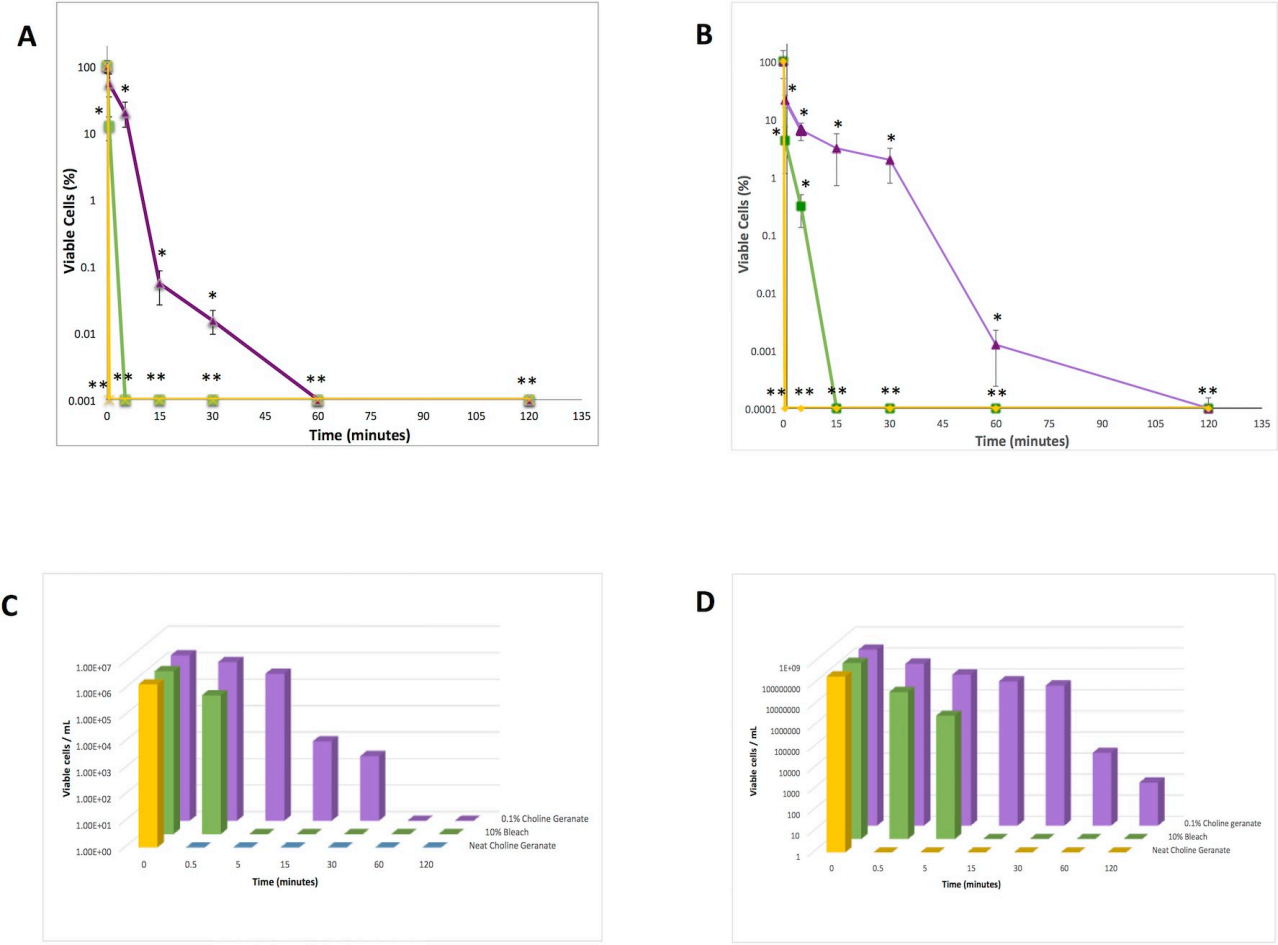
Cellular viability of MSSA biofilms vs time for exposures of neat CAGE (yellow), 0.1% CAGE (purple) and 10% bleach (green). Data is presented as calculated mean cellular viabilities ± standard deviation. A) Percent cellular viability for exposures on 24 hr biofilms, B) Percent cellular viability for exposures on 72 hr biofilms, C) Mean cellular viability vs exposure time for 24 hr biofilms, D) Mean cellular viability vs exposure time for 72 hr biofilms. Errors in a) and b) represent the standard error of the mean for n = 6, and statistical significance for all conditions compared to untreated controls were calculated via the Student’s t-test (* p<0.05, **p<0.01). Calculated means for data points in parts a and b vs time are presented in a logarithmic bar plot in parts c and d for the convenience of the reader. The lowest value on the y-axis of plots a and b represent the limit of detection of the implemented assay.

Neat CAGE was previously shown to reduce biofilms of *Staphylococcus aureus* and *Salmonella enterica* var *typhimurium* over 6 orders of magnitude in a 2 hour exposure.[18] Here, the efficacy of CAGE as an antibiofilm agent was further explored by challenging *in vitro* biofilms of methicillin-sensitive *S*. *aureus* (MSSA) to dilute aqueous solutions of CAGE for various exposure times. Biofilms formed on the polystyrene pegs of minimum biofilm eradication concentration (MBEC) inoculators (Innovotech) were challenged with solutions of CAGE for various time intervals, the agent was then neutralized by washing the biofilms with alternate solutions of dilute ethanol and Cation-adjusted Mueller Hinton broth (CAMHB), and the viable cells enumerated after dispersal via sonication. Neutralization of CAGE is essential for accurate estimation of all time-based biofilm inactivation studies as any residual material from the CAGE challenge is capable of continuing to inactivate bacterial cells after dispersal via sonication. Although prolonged exposure to concentrated solutions of ethanol are well known to affect a general loss of viability in bacteria in culture, we have observed that brief exposures (3 X 10 sec) to the solutions of aqueous ethanol used here are sufficient to remove residual CAGE with minimal impact to cell viability in the biofilm. Representative biofilms of MSSA cultured for 24 hours were subject to a small reduction in bacterial load after the ethanol wash, but the viability in more mature (72 hour) biofilms was observed to vary negligably after this treatment (S4 Fig). The reduction observed in the younger biofilms is likely due to inactivation of peripheral solvent-exposed or adherent cells in these communities which exist to a greater relative extent in biofilms with less mature EPS layers. Importantly, we also note that these brief washes do not sensitize or otherwise introduce bias in the biofilm toward cell killing as cellular viability of ethanol-washed MSSA biofilms exposed to brief (30 second) exposures to 10% bleach do not differ appreciably from comparable biofilms that are not subject to this ethanol wash. The resilience of these representative biofilms toward the ethanol wash was similarly observed for the other strains used in this work. In our assays, we observe neat CAGE to eradicate the biofilm in as little as thirty seconds, even against biofilms cultured for 72 hours (Fig 2). This observation suggests the agent elicits its antibacterial effect upon contact with the biofilm. No viable cells were observed after contact with neat material using the MBEC assay, however we note that the limit of detection of our test (~60 viable cells/mL) is slightly above this observed value.

**Fig 3:**
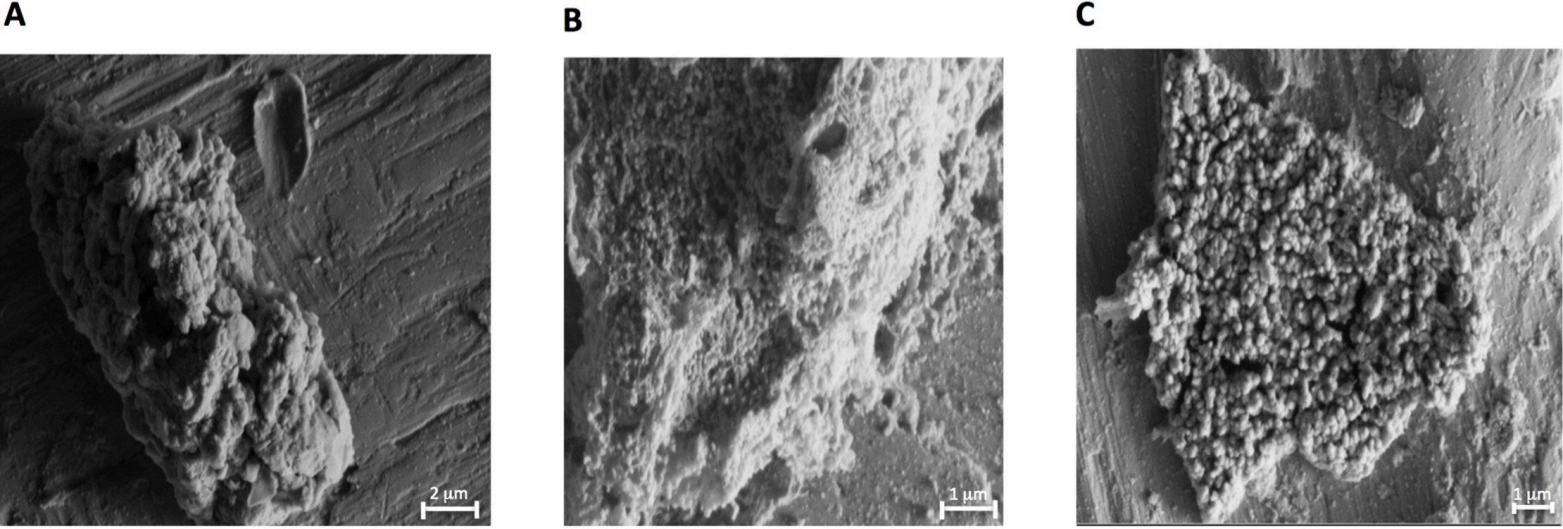
SEM analysis of representative MSSA biofilms. Biofilms were allowed to form onto titanium coupons for 72 hours and subsequently treated with 0.1% CAGE for a) 0, b) 5, and c) 120 minutes.

**Fig 4:**
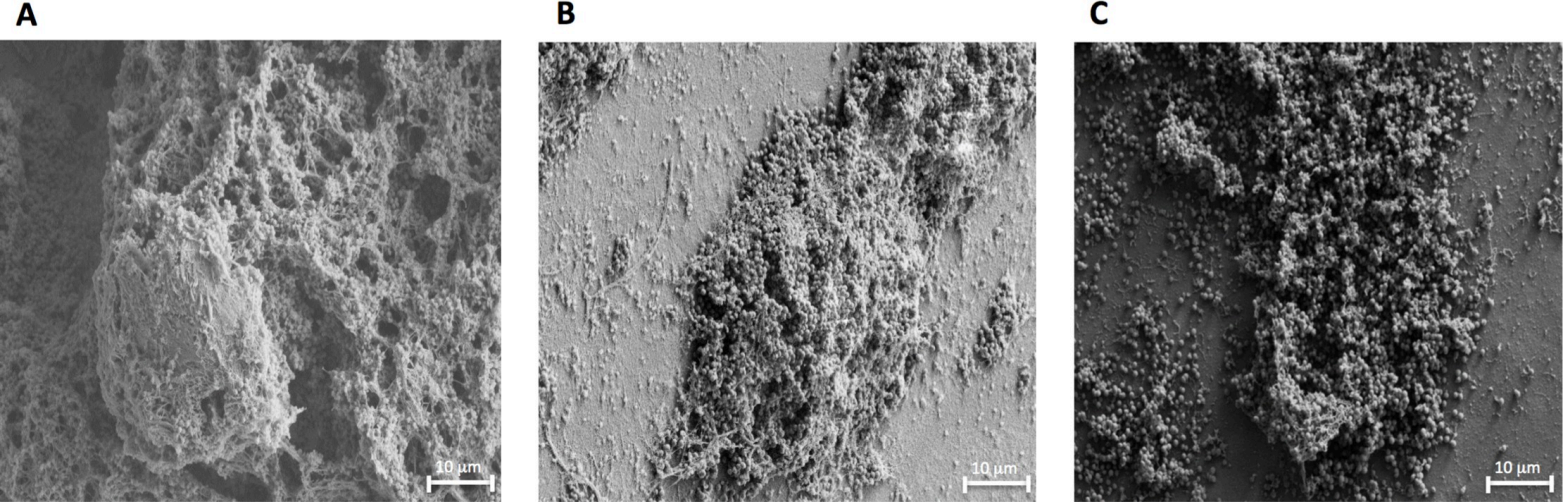
SEM analysis of representative MSSA biofilms. Biofilms were allowed to form onto titanium coupons for 72 hours and subsequently treated with 1% CAGE for a) 0, b) 5, and c) 60 minutes.

A dilute formulation of CAGE (0.1% by volume, in water) was also observed to eradicate biofilms in a rapid and time-dependent manner, with reductions of cellular viability greater than three orders of magnitude realized in 15 minutes against 24 hour old biofilms and 60 minutes against mature 72 hour old biofilms. Mature biofilms are generally regarded to differ from younger biofilms, in part, in the extent and thickness of the protective EPS layer. The rate of cell killing of the biofilms with dilute CAGE solutions were somewhat slower than those observed with 10% bleach, but the rate of its antibacterial action still compares favorably to conventional antibiotics, the majority of which require 24 hours of exposure or more to eradicate comparable *in vitro* biofilms. Interestingly, the rate of viable cell reduction within biofilms exposed to dilute CAGE is observed to be relatively similar regardless of biofilm age. We interpret this finding to infer that the EPS layer (which is ostensibly thicker and more robust in 72 hour biofilms than 24 hour biofilms) does not present as indomitable of a barrier to diffusion of CAGE as it does to other canonical antibiotics. To assess the effect of dilute CAGE solutions on biofilms relative to untreated controls scanning electron microscopy (SEM) was used to image the biofilms and the protective EPS (Figs 3 and 4). Untreated control biofilms (72 hr) were observed to have an amorphous appearance, ostensibly due to the EPS layer of the biofilm. However after five minutes of exposure to 0.1% (v:v) CAGE, coccoid bacterial cells were visually apparent within disruptions in this amorphous layer (Fig 3).

After two hours, the coccus are completely visible throughout the biofilm, indicating significant disruption of the EPS. Similarly, coccoid cells are observed at even shorter times after exposure to 1% (v:v) CAGE (Fig 4).

The SEM data presented here suggests that in addition to its antibacterial properties, CAGE may also aid exposure of the bacterial cells to the environment by disruption of the EPS. The concentrations of CAGE examined here (0.1% and 1.0%, or 2.3 and 23 mM, respectively) were not observed to exert any appreciable toxicity to mammalian keratinocytes. A previous report described that little toxicity to these cells was observable even at the highest concentration of CAGE tolerated by the assay (~50 mM).[15]

### CAGE is broadly effective against biofilms of clinically Isolated ESKAPE and MDR strains

The majority of hospital-acquired infections are attributable to a subset of bacterial species known as the ESKAPE pathogens.[25] These pathogens are both considered among the most prevalent cause of infections to skin and wounds, and are recognized for their burgeoning rates of emergent antibiotic resistance. Thus, while the term ESKAPE is an acronym for these human pathogens, it also harkens to the reason the species are of such particular concern: their ability to ‘escape’ the deleterious effects of antibiotics is on the rise worldwide.[25] We determined the effectiveness of dilute CAGE against 11 isolates of ESKAPE pathogens (including numerous methicillin resistant strains of *S*. *aureus*) from an in-house strain library [26] using the minimal biofilm eradication concentration (MBEC) assay. In each case, both early (24 hr) and mature (72 hr) biofilms were challenged with serial dilutions of CAGE in water and the material displayed broad-spectrum antibiofilm activity against all strains examined (Tables 1 and 2), including several MDR isolates of *S*. *aureus*.

**Table 1:** Observed minimal biofilm eradication concentrations (MBEC) for 30 and 120 minute exposures of dilute CAGE against 24 hour old biofilms of selected ESKAPE pathogens. Each MBEC value represents the largest of three (n = 3) independent measurements.

	30 minute exposure		120 minute exposure	
Species	MBEC (% v:v in water)	MBEC (mM)	MBEC (% v:v in water)	MBEC (mM)
*E*. *cloacae*	0.313	7.11	<0.156	<3.56
*K*. *pneumoniae*	>10	>227.2	0.625	14.2
*P*. *aeruginosa*	>10	>227.2	2.50	56.8
*S*. *aureus (MSSA)*	0.625	14.2	0.313	7.11
*S*. *aureus (MRSA)*	0.625	14.2	0.313	7.11
*S*. *aureus (MRSA)*	0.625	14.2	0.313	7.11
*S*. *aureus (MRSA)*	1.25	28.4	0.625	14.2
*S*. *aureus (MRSA)*	1.25	28.4	0.156	3.56
*S*. *aureus (MRSA)*	2.5	56.8	2.5	56.8

**Table 2:** Observed minimal biofilm eradication concentrations (MBEC) for 30 and 120 minute exposures of dilute CAGE against 72 hour old biofilms of selected ESKAPE pathogens. Each MBEC value represents the largest of three (n = 3) independent measurements.

	30 minute exposure		120 minute exposure	
Species	MBEC (% v:v in water)	MBEC (mM)	MBEC (% v:v in water)	MBEC (mM)
*A*. *baumanii*	1.25	28.4	0.625	14.2
*E*. *cloacae*	1.25	28.4	<0.156	<3.56
*Enterococcus sp*	2.5	56.8	0.625	14.2
*K*. *pneumoniae*	5.0	113.6	1.25	28.4
*P*. *aeruginosa*	>10	>227.2	1.25	28.4
*S*. *aureus (MSSA)*	1.25	28.4	0.625	14.2
*S*. *aureus (MRSA)*	2.5	56.8	0.313	7.11
*S*. *aureus (MRSA)*	5.0	113.6	0.625	14.2
*S*. *aureus (MRSA)*	2.5	56.8	0.313	7.11
*S*. *aureus (MRSA)*	1.25	28.4	0.313	7.11
*S*. *aureus (MRSA)*	2.5	56.8	0.625	14.2

CAGE demonstrated an ability to rapidly and effectively eradicate both early and mature biofilms of all pathogens examined. As we have previously observed, the biofilms grown for 24 hours were observed to be more susceptible to eradication than those cultured for 72 hours at both of the examined durations of exposure, although slightly higher concentrations of CAGE were required to completely eradicate the biofilms of all strains after 30 minutes of exposure relative to those required for eradication at 120 minutes. Isolates of two species (*Enterococcus* sp and *Acinetobacter baumanii*) were not observed to form robust biofilms in this assay after 24 hours, and thus the activity of CAGE against them was determined upon 72 hour old biofilms only. The MBEC values observed against the examined MRSA strains were not appreciably different than that observed for a methicillin-sensitive *S*. *aureus* isolate, which shows CAGE is effective against this species in general but also importantly infers that little cross-resistance to CAGE is facilitated by drug resistance mechanisms that exist within these organisms. Furthermore, we also tested CAGE against a recombinant strain of *E*. *coli* reported to be resistant to ionic liquids. [27, 28] *E*. *coli* BL21 DE3 eilA/R harbors a plasmid encoding the *eilA/R* genes that encode a transmembrane efflux pump and regulatory protein, respectively. The *eilA/R* system was first identified in an ionic liquid tolerant bacterium *Enterobacter lignolyticus* [28] and the expression of EilA/R has been reported to engender broad resistance toward a number of imidazolium based ionic liquids.[27] To our knowledge, the EilA/R efflux system is the only identified ionic liquid resistance mechanism identified to date. The MBEC (2 hour exposure) of CAGE towards 72 hours biofilms of *eilA/R* expressing strains (2.5% v:v; 56.8 mM) is not appreciably different than the MBEC against native *E*. *coli* BL21 DE3 (1.25%; 28.4 mM), which also illustrates little cross-resistance to CAGE is facilitated by EilA/R. It should be noted that in strains containing *eilA/R*, efflux of imidazolium and pyridinium cations is mediated by the transmembrane transporter EilA, and expression of the *eilA* gene is affected by an imidizolium/pyridinium cation induced interaction with the genetic regulator EilR [27]. At this time we cannot conclude if the effectiveness of CAGE against this strain is primarily due to a limited interaction with EilR or an ineffective efflux by EilA, but either case would represent a desirable pharmacological property of new materials to target any strains demonstrating emergent ionic liquid resistance.

### Molecular modeling predicts interaction of geranate and geranic acid with both leaflets of a model membrane

**Fig 5:**
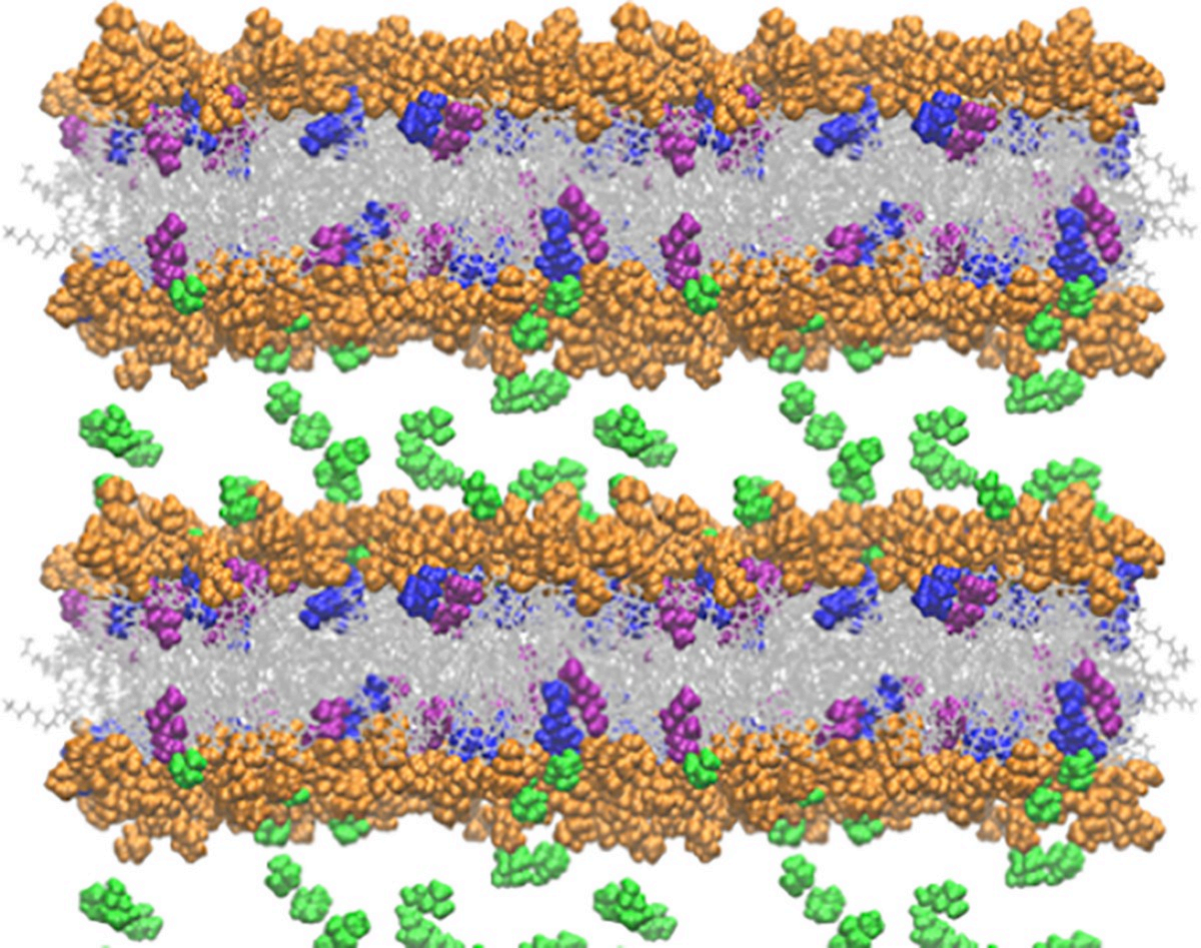
Static representation of model POPE membranes in the presence of CAGE. The lipid head groups are depicted in orange, the lipid tails in grey, cholinium cation in green, geranate in purple, and geranic acid in blue. Water molecules are omitted for clarity. Several periodic images are shown to make the long ranged structure clearer.

While our results show CAGE to be broadly effective against biofilms of bacterial pathogens, the means in which it inactivates the underlying cells is currently less clear. Information on the mechanism used by CAGE to kill pathogens is invaluable for efforts to synthesize second generation materials with improved potency. In order to illuminate the potential antibacterial mechanism of action, molecular simulations of aqueous CAGE and a palmitoyloleoyl phosphatidylethanolamine (POPE) membrane were conducted to assess predicted interactions between the two species. Bacterial inner membranes are known to be relatively rich in POPE fatty acids, and thus the POPE membrane was chosen as a convenient model of a bacterial membrane.[29] Simulations indicate that both the geranate anion and the protonated (neutral) geranic acid components of CAGE readily insert into the POPE membrane. The geranate anion was observed to align with the polar phospholipid head groups of the leaflet and the lipophilic alkyl moiety aligning with the hydrophobic tails of the fatty acids in the bilayer. Geranic acid was found to interact with the hydrophobic environment of the membrane, but this neutral species was not observed to align with the amphiphilic fatty acids in the same orientation as the negatively charged geranate anion. In the simulation, geranic acid was found to readily fluctuate between both leaflets of the nascent membrane as the bilayer developed, whereas geranate remained in a leaflet as the bilayer stabilized (Fig 5).

In all simulations, the cholinium cation was present within the water layer and found to transiently associate with the negatively charged lipid bilayer head groups on the leaflet surface.

### CAGE is predicted to facilitate membrane thinning, not membrane disruption, in a model membrane

Our observation of the interactions of the geranate anion interactions within the POPE membrane were consistent with previous studies with imidazolium ionic liquids. Briefly, the lipophilic ions were similarly found to align with the composite fatty acids and preferentially populate the membrane leaflet. However, models of imidazolium ionic liquid interactions predicted an overload of the lipophilic component into the outer leaflet, which subsequently caused membrane deformation, strain and eventually membrane (and cellular) lysis.[30] This general antibacterial mechanism of action is also shared by other cationic antiseptics.[31] In contrast with the imidazolium ionic liquids, however, bacterial cell disruption was not observed upon CAGE-mediated eradication of pathogenic microbes.[15] A variety of molecular mechanisms have been proposed to account for the ability of amphiphilic compounds to interact with lipid bilayers in order to ultimately exert an antibacterial effect.[32, 33] Destabilization of the bilayer through membrane thinning (which ultimately results in cell death) has been observed for many cationic peptides.[34–38] Consistent with these, our models also predict that insertion of the geranic acid/geranate moieties of CAGE into POPE membranes results in a reduction of bilayer thickness and an increase in area per lipid (Table 3).

**Table 3:** Effect of CAGE concentration on POPE area per lipid and bilayer thickness.

	Area per lipid (Å^2^)	Bilayer thickness (Å)
0 IL	66.8±1.1	42.0±0.6
8 IL	69.9±1.3	41.1±0.7
16 IL	72.6±1.4	40.4±0.7
32 IL	80.0±1.2	38.9±0.4
64 IL	93.5±1.2	36.0±0.4

Table 3 shows that the lipid groups occupy increasingly larger areas and that the bilayer becomes thinner as the CAGE concentration increases, which may provide verifiable hints of the antibacterial effects of CAGE on bacterial cell membranes. Many antimicrobial peptides that are known to modulate membrane thickness exert their antibacterial action through cellular lysis. [35, 37, 39] Interestingly, three of the antimicrobial peptides examined by Grage et al were observed to affect a thinning of the examined membrane.[35] Similarly, our simulations show membrane thinning to a POPE bilayer, which in turn suggests the thinning event is likely sufficient to elicit a biological effect. While CAGE exposure is not observed to lyse cells outright, it is not difficult to imagine that the general disruption to membrane homeostasis and important membrane localized enzymes by this thinning would be sufficient to inactivate cells.

### Geranic acid provides an important contribution to antibacterial activity of CAGE

By virtue of its ability to populate either leaflet, our model also suggests that the neutral geranic acid species in CAGE enables the lipophilic isoprenoid moieties of CAGE to populate the membrane as a whole, and thus may play a role in potentiation of the agent when it is formulated as an IL/DES (i.e cholinium:geranate:geranic acid) relative to the antibacterial properties it displays upon formulation as a salt (cholinium:geranate). This hypothesis from the computational model is supported experimentally through analysis of MBEC values for each respective formulation. CAGE formulated as a 1:2 ratio of cholinium cation:geranate/geranic acid (MBEC = 0.625%; 14.2 mM) is observed to be roughly 13-fold more effective than formulations of CAGE as a 1:1 ratio of cholinium cation:geranate (MBEC = 5% v:v; 184.9 mM) in 2 hour challenges against 72 hour old MSSA biofilms. Direct assessment of the antibiofilm activity of protonated geranic acid itself was not determined as the acid is sparsely soluble (<50 mM) in aqueous (unbuffered) conditions, even in the presence of solubilization enhancers such as DMSO or polysorbate based detergents (e.g. Tween 80) at the maximum final concentrations recommended by CLSI (1% and 0.1%, respectively).[40] The concentrations of DMSO or Tween 80 required for full solubilization of the acid in water were incompatible with subsequent analysis in the MBEC assay. Further evidence for the importance of the protonated species was observed in tests with equilibrium mixtures of geranic acid and geranate ion, however. Buffered aqueous solutions of the geranic acid-geranate equilibrium (10% v:v solutions in 50 mM HEPES with 1.5% Tween 80) elicited an antibacterial effect against 24 hr *S*. *aureus* biofilms that was at least four times more pronounced at pH 6.4 (observed MBEC = 2.5% v:v; 144 mM) than at pH 8.4 (observed MBEC > 10%; >577 mM). No appreciable change in biofilm viability relative to controls was observed upon exposure to buffered solutions in the absence of CAGE. While the equilibrium exists at both examined pH values, at pH 6.4 the protonated species is two orders of magnitude more abundant in solution than it is at pH 8.4. The increased potency of the more acidic solution also supports our hypothesis that the neutral geranic acid species in CAGE plays an important role in the antibacterial action of the composite material.

### Distribution of moieties of CAGE within an aqueous bilayer model

**Fig 6:**
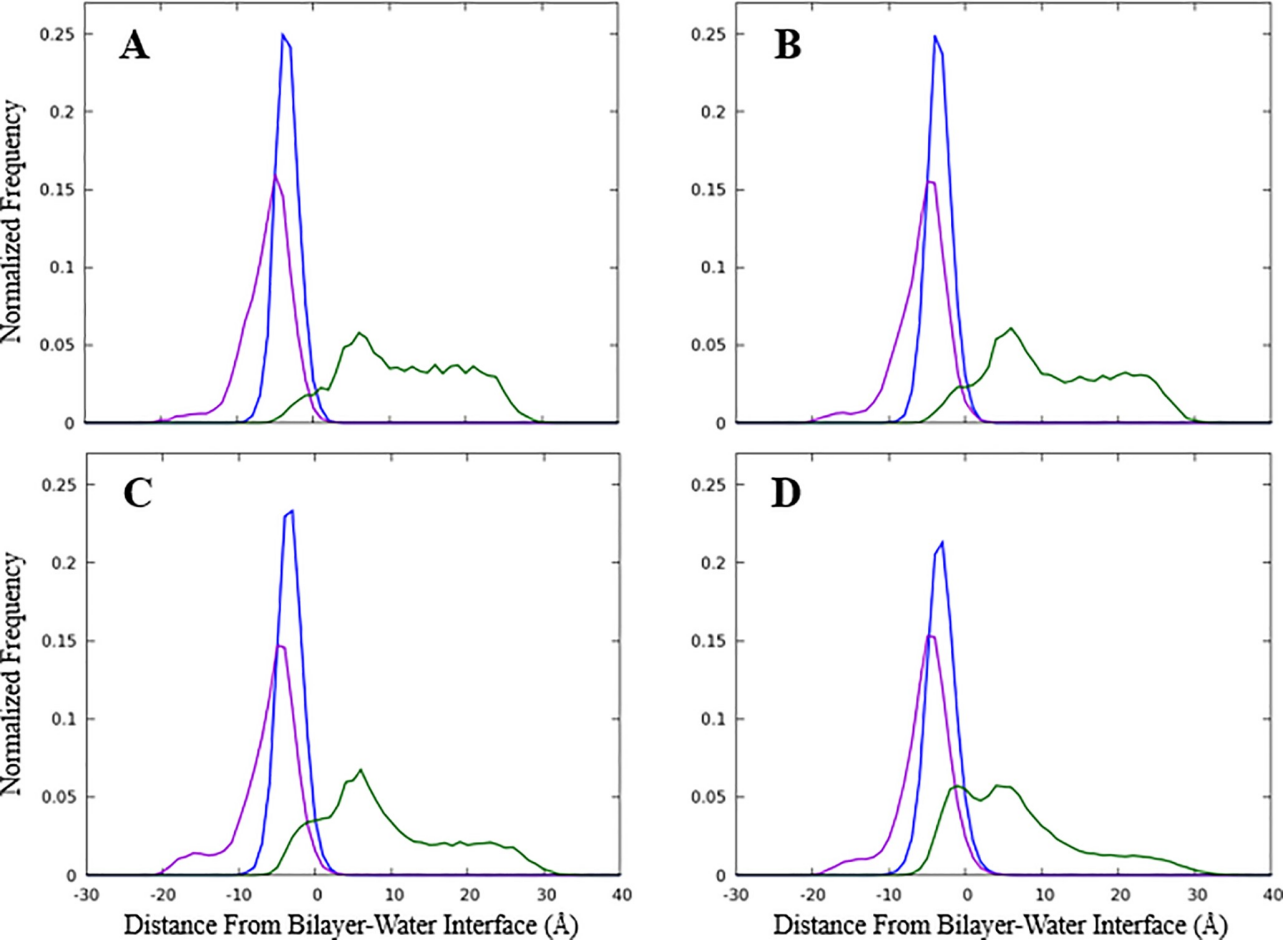
Probability distribution of CAGE components into a model membrane. Simulations defined the probability distribution of components perpendicular to the average bilayer surface with A) 8 CAGE, B) 16 CAGE, C) 32 CAGE, or D) 64 CAGE components added. The cholinium cation density is shown in green, geranate in blue, and geranic acid in purple. The zero point is the average position of the phosphorus atom from each lipid in the leaflet, where negative values are within the bilayer and positive values are in the aqueous phase. Results from each leaflet are averaged to improve statistics.

While our modeling studies predict a number of phenomena that could help explain the potent bactericidal activity of CAGE, the root of its selectivity toward bacterial pathogens relative to human cells remains less clear. Mammalian cells have a higher percentage of phosphatidylcholine within their membranes than prokaryotes, and we have postulated that CAGE, which has some structural similarity to phosphatidylcholine, does not enable deformations of membrane that contain high percentages of this phospholipid to the same extent it does for those predominantly composed of phosphatidylethanol. (17) Although the dynamic modeling data suggests that, at least on an atomistic level, the ion pairing within CAGE is largely not maintained as the agent embeds into the membrane surface in an aqueous environment, the probability distribution of the groups indicates some pairing may be possible. Fig 6 shows the average distribution of the CAGE components perpendicular to the bilayer interface. The cholinium cation is found primarily in the aqueous phase, while the geranate anion and geranic acid molecule embed into the bilayer.

Interestingly, there is significant overlap between the cholinium and geranate densities, indicating that ion pairing can occur even though the cations and anions partition into different portions of the system. It is important to note that the dynamic simulations predict molecular associations originating from a completely disordered state. In this, all molecules in the system, including CAGE, water, and POPE diacylglycerols begin from a random assortment in bulk solvent and molecular interactions are subsequently measured as a function of time. Conversely, in the *in vitro* experiments, neat CAGE is diluted in water and then added to the biofilms in which the relevant membranes are located. Recent investigations indicate the physicochemical factors that govern the interaction of CAGE in solution are more complex than those for most simple organic salts. Preparations of CAGE with varying molar equivalents of geranic acid display complex coordination structures as dilute solutions in d_6_-DMSO,[41] and preliminary investigations in our lab indicate unusual solution properties in water at some CAGE concentrations. Future work will strive to obtain a more detailed understanding of the intermolecular interaction of CAGE moieties both as neat material or dilute solutions in water, as well as the interactions of CAGE with the aqueous medium itself. In any case, both the experimental and computational results presented here implicate the geranic acid species, and to a lesser extent the geranate anion, as the pharmacologically relevant antibacterial component of CAGE.

**Fig 7:**
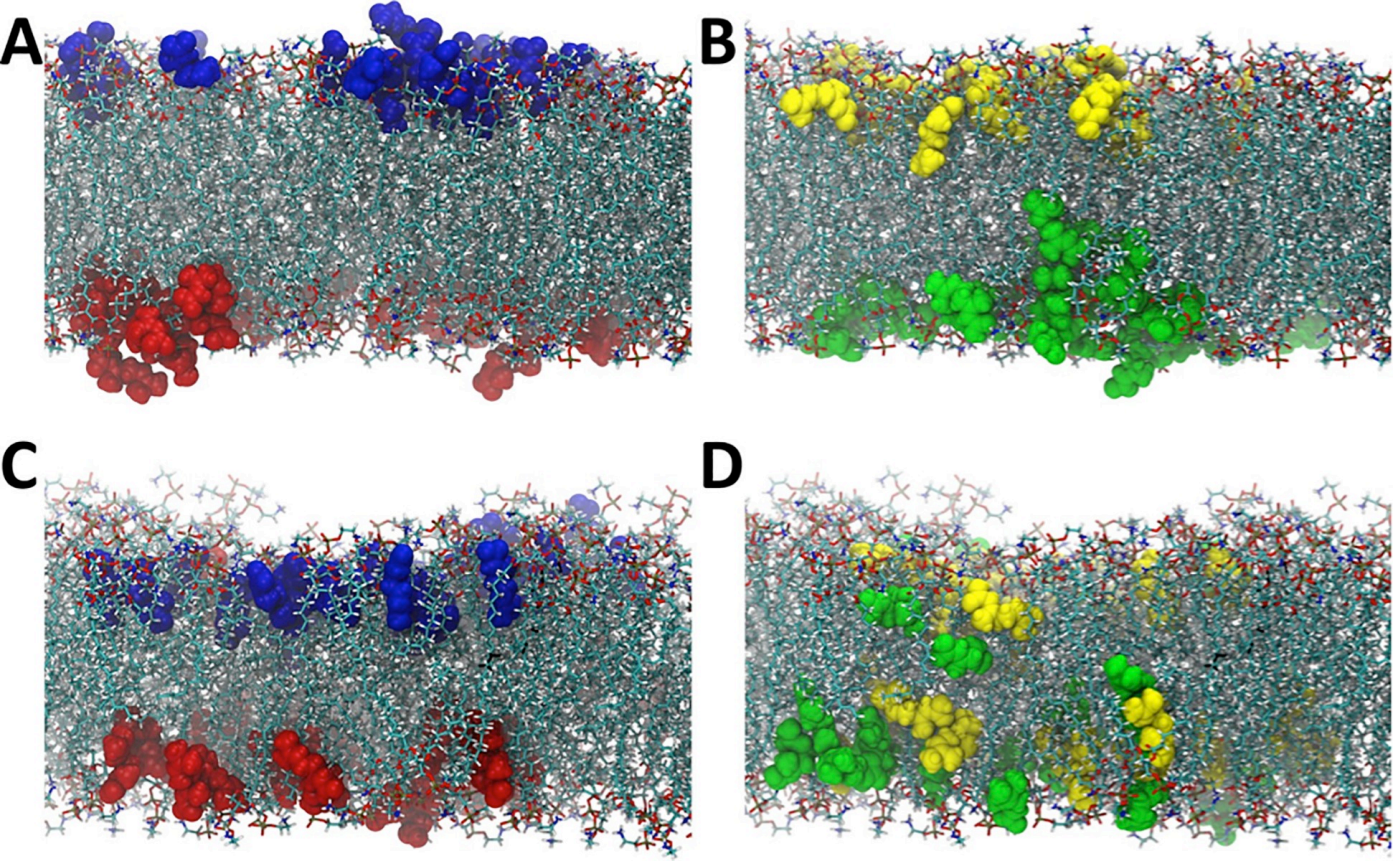
CAGE migration over time within leaflets of the membrane bilayer. Snapshots from the symmetric bilayer simulation with the CAGE components colored according to the leaflet they begin in. Panel A depicts an early production configuration, which shows geranate anions are highlighted in the top leaflet in blue and in the bottom leaflet in red and panel C shows the final configuration after 130 ns of simulation with anions all remaining within the leaflet that they begin. Similarly, panels B and D show geranic acid molecules at the beginning (panel B) and the end (panel D) after 130 ns of the production phase. The yellow and green molecules indicate molecules that begin in the upper (yellow) or lower (green) leaflets. The geranic acid is found to more freely traverse across the bilayer and randomize during our simulation as indicated by the yellow and green molecules randomizing at the end of the production phase.

In order to further explore the behavior of geranate and geranic acid in a lipid bilayer, we carried out a simulation in which we began with these molecules embedded in a POPE membrane system. To that end, initially the geranate and geranic acid molecules were dispersed evenly into the upper and lower leaflet of the POPE membrane. In Fig 7A we show the first frame of the production phase of simulation (e.g., subsequent to minimization, heating, and equilibration phases), with geranate molecules that are in the upper leaflet colored in blue, and geranate molecules in the lower leaflet colored in red.

Similarly, in Fig 7B, the geranic acid molecules in the upper leaflet are colored in yellow and those in the lower leaflet are colored in green, again for the first frame of the production simulation. Fig 7C and 7D demonstrate what happens to these geranate and geranic acid molecules after 130 ns of unrestrained simulation. Specifically, we observe that the geranate molecules remain in the leaflet in which they start, aside from a single geranate molecule that moves from the bottom leaflet to the top leaflet (Fig 7C). It should be noted that this single geranate molecule does not transfer leaflets by moving through the membrane center, however, but rather exits the lower leaflet into the aqueous phase (water and cholinium molecules not shown for clarity) and crosses the simulation cell periodic boundary to then embed into the upper leaflet. Therefore, no geranate molecules are observed to transit across the interior of the membrane between the two leaflets over the simulated timescale. This is in stark contrast to what is observed for the geranic acid molecules in our simulation. Geranic acid molecules mix readily between the leaflets over the 130 ns simulation, and traverse back and forth across the membrane center (Fig 7D). This is demonstrated clearly in Fig 7D by the mixing of yellow and green colored geranic acid molecules in both of the membrane leaflets. This robustly demonstrates one of the central observations from molecular dynamics simulation of CAGE + membrane systems that cannot be observed from experiment alone, which is that the geranate ions predominantly remain associated with the phospholipid heads, remaining in a single monolayer, while geranic acid molecules are much more free to move through the hydrophobic bulk of the membrane. Chemically this can be related to the fact that the geranic acid molecules are protonated, and therefore charge neutral, making the hydrophobic bulk a more hospitable environment for geranic acid compared to the negatively charged geranate molecule. This observation underscores the importance of the geranic acid component of the CAGE solvent, which is freely able to navigate the lipid bilayer, unlike geranate, and also unlike cholinium, which remains in the aqueous phase. These observations are consistent with the behavior that was recently reported using a coarse-grained model (the MARTINI model), [42] however our simulations provide increased atomistic detail into the process of CAGE transport in a lipid bilayer compared to coarse-grained simulations. Furthermore, these all-atom simulations are the first of their kind for CAGE in a lipid environment, and directly demonstrate that starting from the basic physics of an all-atom model, over relatively reasonable timescales, that phase separation and transport properties can be inferred while making only limited approximations in the model itself.

## Discussion

In this work, we have conducted a detailed assessment of the antibiofilm properties of CAGE against a suite of clinically relevant ESKAPE pathogens, and shown that it is capable of rapidly eradicating mature biofilms, even as dilute formulations. Our data demonstrates that CAGE begins to disrupts the EPS layer of biofilms and destroys the underlying cells upon contact, and its potency and time required for full biofilm eradication compare favorably to literature values reported for other common standard-of-care topical antiseptics. Established methicillin-resistant strains as well as those demonstrating resistance to imidazolium-based ionic liquids were both effectively eradicated by CAGE, indicating the risk of cross-resistance to CAGE by existing resistance mechanisms is low. Unlike many IL and cationic surfactants, which insert into the leaflet of the membrane bilayer they encounter, molecular modeling coupled with *in vitro* antibiofilm testing suggests CAGE exposure facilitates saturation of the entire bilayer of the membrane with geranic acid. Thus, our work points to geranic acid, and to a lesser extent geranate ion, as the likely antibacterial component of CAGE and suggests thinning of the membrane and greater average spacing between the lipid headgroups, along with a concomitant disruption of homeostasis in the bacterial membrane as a likely mechanism of antibacterial action for CAGE. Although CAGE demonstrates broad-spectrum antibiofilm activity, we note that its *in vitro* potency is lower than that reported for compounds considered best in class antibiofilm agents designed for systemic antibiotic therapy.[43, 44] CAGE is superior to these aforementioned materials, however, in its ability to penetrate skin, as few, if any, antibiotics designed for systemic delivery are capable of transdermal penetration in the absence of chemical augmentation. This property enables CAGE to serve as a topically administered therapy to effectively treat subdermal infections, which is a feature rarely observed for canonical antibiotics or antiseptics. The ability to penetrate skin is a particularly important property in the battle against wound infections as the physical location of the invading microbial biofilms are typically within the dermal layer on the periphery of the wound itself. In addition to its own antibiofilm activity, CAGE has long been recognized for its ability to facilitate transdermal delivery of other co-administered antibiotics. Thus, while the potency of CAGE may seem modest when compared to best in class systemic agents, its unique molecular abilities afford it a favorable potency relative to comparable literature values provided for many of the topical antiseptics in current clinical use [16, 45–47], and commercial products containing chlorhexidine (e.g. ChloraPrep) or povidone-iodine antiseptic wipes commonly contain the active ingredients at 2–5% or 5–10% (w:v), respectively. Similarly, in addition to cleansing tissue, the rapid reduction in existing biofilms of concentrated solutions of CAGE may make it a useful material for decontaminating indwelling medical devices via antibiotic-lock therapy.[48] However before any medical implementation of CAGE may proceed, a precise estimate of its toxicity on mammalian systems must commence. To date, a complete pre-clinical assessment of the toxicity profile of CAGE has not been provided, however, this assessment is the subject of ongoing work in our team.

As noted previously, there is a pressing societal need for new and effective antiseptic materials. CAGE was developed to address this need, but due to its ability to penetrate skin, more recently it has also been developed as a unique vehicle for oral delivery of insulin.[41, 49, 50] As such, the broad survey of the effects of CAGE on microbes in sessile culture presented here is expected to be desirable both to determine its potential for medical/antiseptic use as well as to estimate its effect on communal intestinal flora. Furthermore, the mechanistic insight into of the mode of cell inactivation facilitated by CAGE we have provided is expected to be very valuable to the development of second generation IL/DES with improved antibacterial or physicochemical properties and safety profiles.

## Materials and methods

### General procedures

All chemical reagents were obtained from standard commercial suppliers. All antibiofilm assays were conducted on biofilm inoculators (Innovotech) following the manufacturer’s recommendations with minor modifications. Antimicrobial data was collected on a BioTek Synergy H6 plate reader to measure absorbance at 600 nm. Clinical isolates of all pathogens were from an in-house strain library maintained at NAU. All microbial manipulation of pathogenic bacteria was conducted in a certified biosafety level 2 laboratory while following all associated safety protocols. The term MBEC in this manuscript refers to the definition suggested by the assay’s manufacturer and within an approved international standard,[51] which is the minimal concentration of compound required to result in no measurable cellular density in a solution of disrupted biofilms that had been subject to a 24-hour post-sonication outgrowth. All MBEC assays were performed on a minimum of three independent biological replicates, each of which contained four experimental replicates per test. MBEC values represent the highest determined from three biological replicates in cases where MBECs varied by 2-fold.

### Synthesis of CAGE

CAGE was synthesized in a manner analogous to that described in Zakrewsky *et al*. [19] Briefly, one equivalent of choline bicarbonate (80 wt% solution, 10.8 g, 0.052 mol) was added, dropwise, to a 50 mL round bottom flask containing two equivalents of recrystallized geranic acid (18.8 g, 0.112 mol). The mixture was covered with foil and stirred at room temperature until evolution of CO_2_ had ceased. At this time, the mixture was dried in vacuo at 50°C for 12–16 hours to dehydrate the DES. A total of 21.4 g (95%) of CAGE was obtained as a light yellow oil. All physical and chemical characterizations matched previously published reports. It is worth noting that a multiplet at 2.60 ppm is visible to a varying extent based on reagent supplier (unpublished obsd) or choice of NMR solvent (T. Welton, personal communication). It appears as a minor contaminant in the spectrum reported here. ^1^H NMR (CDCl_3_): δ 5.52 (s, 2H), 5.03 (t, J = 7, 2H), 3.83 (t, J = 6.3, 2H), 3.38 (t, J = 5.1, 2H), 3.08 (s, 9H), 2.02 (m, 8H), 1.95 (s, 6H), 1.69 (s, 2H), 1.60 (s, 6H), 1.53 (s, 6H); ^13^C NMR (DMSO-d_6_): δ 169.9, 149.7, 131.5, 124.3, 122.2, 67.6, 55.5, 53.6, 32.75, 26.9, 25.9, 18.1, 17.9.

### Biofilm cultivation using MBEC biofilm inoculators

Individual bacterial colonies were isolated from by Luria-Bertani (LB) agar streak plates grown from frozen 30% glycerol stocks from each examined strain. A single colony from the plate was used to inoculate Cation-Adjusted Mueller-Hinton broth (CAMHB, 5 mL) and incubated overnight at 37°C with shaking (225 rpm). The resulting confluent culture (50 mL) was used to inoculate fresh CAMHB (5 mL) and was again incubated with shaking (37°C; 225 rpm) until mid-logarithmic growth was observed (~OD_600_ = 0.4–0.6). Growth rates for each strain were observed to vary by species, but mid-logarithmic growth was typically attained in approximately 3–4 hours. At this time, the sample was diluted (1:50 v:v ratio) into fresh CAMHB and 150 **μ**L was immediately placed into each of the wells of the 96 well plate of the biofilm inoculator (Innovotech). The pegged lid of the MBEC biofilm inoculator was placed upon the plate and the edges sealed with parafilm to prevent evaporation. The apparatus was then incubated with shaking (37°C; 180 rpm) overnight at which time the lid was either immediately subjected to antimicrobial assays (for studies with 24 hour biofilms) or transferred to a new sterile 96-well plate filled with CAMHB to continue growth. For 72-hour biofilms, the procedure was repeated twice further. Culture of all organisms in CAMHB was chosen in order to align with standards for antibiotic testing in planktonic culture set forth by the Clinical and Laboratory Standards Institute, however in our hands biofilm densities for all examined strains varied negligibly between this medium and other common media (LB, TSB, etc). Average biofilm densities in CAMHB are provided in the supplementary information (S5 Fig)

### MSSA biofilm cultivation using the CDC biofilm reactor

Use of the CDC biofilm reactor followed the manufacturers protocol. Briefly, individual MSSA colonies were obtained from an LB agar streak plate, and a single colony was used to inoculate Luria-Bertani broth (LB, 5 mL) and incubated overnight at 37 °C with shaking (225 rpm). The resulting confluent culture (1 mL) was used to inoculate fresh LB (400 mL) within the biofilm reactor chamber. The bioreactor was pre-loaded with seven titanium coupons, each of which had been pre-cleaned with acid (2 M HCl) for two hours, washed with water and air-dried. The reactor was incubated at room temperature for 24 hours with stirring (125 rpm). At this time, fresh LB was introduced into the chamber via peristaltic pump (1.5 mL/min) with stirring (125 rpm), and spent media removed from the chamber into a waste container. After a further 24 hours, the bioreactor was disassembled, and sterile forceps were used to carefully remove the coupons to avoid disruption of the biofilms on the surfaces. Coupons were placed into sterile petri dishes and processed immediately.

### MSSA biofilm time-kill assays

Biofilms of MSSA were formed biofilm inoculators for either 24 or 72 hours as previously described. A total of six inoculators per biofilm age were used in each assay. Prior to challenging with CAGE, all cultivated biofilms were rinsed by immersion of the pegged lid of the inoculator (3 X 10 sec) into a sterile 96-well plate containing fresh CAMHB (200 **μ**L) to remove any planktonic cells. At this time, all lids were transferred to fresh 96-well microtiter plates containing challenges of neat CAGE, 2.2 mM aqueous CAGE (0.1% v:v), 10% aqueous bleach, and fresh CAMHB (200 **μ**L per well). Biofilms were removed from the challenge solutions at various time intervals over the course of two hours. Upon removal, each inoculator lid was immediately rinsed by immersion into 50% aqueous ethanol (3 X 10 sec) followed by rinsing with fresh CAMHB (3 X 10 sec) to neutralize any residual CAGE associated with the biofilms. The inoculator lid was then transferred into a new sterile 96 well plate containing fresh CAMHB (175 **μ**L/well), covered with parafilm and biofilms dislodged from the pegs via sonication (Misonix S3000, power 0.5, duty cycle 3 sec, 60 minutes). Viable cells were then quantified by dilution and enumeration on solid medium. Enumeration was performed by dispensing small (3 X15 **μ**L) droplets of the disrupted biofilms in solution or of subsequent dilutions onto a solid CAMHB-agar plate. Given this implementation of the quantitative assay, we estimate that the limit of detection in this case is equivalent to approximately 60 cfu/mL in an undiluted sample (~1 emergent colony after incubation in a 15 **μ**L droplet). All assays contained a minimum of three experimental replicates per biological replicate and were performed on a minimum of two biological replicates (n = 6).

### MBEC analysis

Each MBEC inoculator containing 24 or 72 hour old cultivated biofilms was rinsed by immersion of the pegged lid (3 X 10 sec) into a sterile 96-well plate containing fresh CAMHB (200 **μ**L) in order to remove any planktonic cells. The lid was then transferred to a second 96-well plate containing two-fold serial dilutions of CAGE in CAMHB (from 227 mM to 3.56 mM; 200 **μ**L/well), covered with parafilm, and incubated with shaking (37 °C; 110 rpm) for either 30 or 120 minutes. The serial dilution plate also contained wells of fresh CAMHB and 10% bleach as positive and negative controls, respectively. At this time, the lid was removed and rinsed by immersion into 50% aqueous ethanol (3 X 10 sec) followed by rinsing with fresh CAMHB (3 X 10 sec). The inoculator lid was then transferred into a new sterile 96 well plate containing fresh CAMHB (175 **μ**L/well), covered with parafilm and biofilms dislodged from the pegs via sonication (VWR Aquasonic P250D, power 4, 10 minutes). At this time, the pegged lid of the inoculator was removed from the 96-well plate, replaced with a standard flat lid, and incubated for 24 h at 37 °C. Bacterial growth in the wells was assessed via absorbance at 600 nm using a microplate reader (Biotek Epoch 2). The MBEC value was defined as the lowest concentration of CAGE required to effect a complete inhibition (OD600 of 0.1 or less) of bacterial growth in this plate after the post-sonication 24 hour incubation period.[51] Each MBEC inoculator contained a minimum of four experimental replicates per biological replicate, and all MBEC assays were performed on a minimum of three biological replicates (n = 9).

### SEM imaging of CAGE challenged MSSA biofilms on coupons

Coupons containing cultured biofilms were rinsed with PBS (3 X 5 mL) to remove planktonic cells and residual media. At this time, the coupons were immersed into 0.1% CAGE (v/v) in water, and were removed and rinsed with 50% aqueous ethanol (1 X 5 mL) followed by sterile deionized water (3 x 5 mL) at various time points (0.5–120 min). An untreated control coupon was processed similarly to the CAGE challenged biofilms. After the CAGE challenge, all coupons were soaked in 2.5% (v/v) glutaraldehyde in PBS for 2 hours at 4°C ^49^, and rinsed with PBS (10mL). All biofilms were dehydrated using ethanol, mounted onto stubs with carbon tape and coated with a gold/palladium alloy using a Denton Vacuum Desk II for ten seconds. All samples were imaged using a Ziess Supra 40vp scanning electron microscope at a voltage of 5 kV.

### SEM imaging of CAGE challenged MSSA biofilms on inoculator pegs

Biofilm inoculators containing 72-hour old MSSA biofilms were grown as described previously at which time individual pegs were removed from the inoculator with forceps. Individual pegs were rinsed with PBS (3X 0.2 mL) and immersed in 1.0% (v:v) CAGE in water. Individual pegs were removed and rinsed with ethanol (1 X 0.2 mL) followed by sterile deionized water (3 x 0.2 mL) at various time points (0.5–60 min).

Following the rinse, the pegs were fixed with 2.0% glutaraldehyde solution in a sodium cacodylate buffer (0.15 M, pH 7.4) for 24 hours at 4° C. The pegs were then secondarily fixed using 1.0% osmium tetroxide in a 0.15% sodium cacodylate buffer. The pegs were subsequently submersed in a 0.15% cacodylate buffer for one hour to remove excess fixative. The samples were dried via ethanol rinses and were mounted on stubs using double sided carbon tape and coated with a gold/palladium alloy using a Denton Vacuum Desk II for ten seconds to ensure that small structures would remain visible. All samples were imaged using a Zeiss Supra 40VP scanning electron microscope at ~1500X magnification at a power level of 3kV.

### Statistical analysis

All cell enumeration data are reported as the mean ± standard deviation. Statistical significance was determined with a two-tailed, unpaired Student’s t-test. The level of significance was set at p < 0.05.

### Computational methods

The simulation results presented here employ POPE lipids as a model for bacteria biological membranes to elucidate the antibiotic action of CAGE. There are two simulation studies described here: lipid bilayer self-assembly with CAGE and symmetric bilayers with embedded CAGE. Molecular dynamics simulations were performed using the Amber16 molecular dynamics software package. [52] Simulation workflows have been automated using the Python-based parallel scripting library Parsl and are available upon request. [53] The AmberTools program tleap was used to parameterize the system.[52] The interactions were described using the Lipid14 for lipids, [54]TIP3P for water,[55] and the general Amber force field for the CAGE components[56]. The partial charges for the CAGE components were assigned using the AM1-BCC model as implemented in the AmberTools utility antechamber. In each system, the number of water molecules was selected so that the bilayer was in the liquid phase. Except for some stages of preparation and equilibration, the temperature was held constant at 310 K with a Langevin thermostat using a collision frequency of 1.0 ps^-1^. For NPT simulations, a Berendsen barostat was used to maintain constant pressure with a relaxation time of 2 ps. Bonds including hydrogen were maintained at a constant length with the SHAKE algorithm so that a time step of 2 fs could be used, except where otherwise specified for equilibration. A 10 Å cutoff for non-bonded interactions was used throughout, which is prescribed for the use of the Lipid14 force field.[54]

### Bilayer self-assembly simulations

Generation of lipid bilayers generally followed a scheme similar to that of Skjevik and coworkers.[57] Random initial configurations of all components were constructed using Packmol. [58] Each simulation consisted of 128 lipid molecules, 6398 water molecules, and the indicated number of CAGE components in a 1:1:1 cholinium:geranate:geranic acid ratio. The simulations are labeled according to the number of CAGE components added, so, for example, the ‘8 IL’ simulation has eight of each cholinium, geranate, and geranic acid. The simulations were performed at a temperature of 310 K. Long range electrostatic interactions are calculated using the particle mesh Ewald algorithm, as implemented in Amber. These configurations were first minimized using a five part steepest descent procedure: minimization was run four times for 10 steps followed by one minimization of 10,000 steps. Equilibration employed isotropic pressure coupling and consisted first of 10 ns of with a 0.5 fs time step and second of 10 ns with a time step of 1 fs. The production simulations used anisotropic pressure coupling.

### Symmetric bilayer simulations

A POPE lipid bilayer with geranate and geranic acid already embedded in the membrane was constructed using the program Packmol. [58] Each of the membrane leaflets contained 64 lipid molecules. Additionally, 32 geranate molecules, and 32 geranic acid molecules were also interspersed into each leaflet. A total of 7,808 water molecules were distributed on either side of the membrane, and 64 cholinium molecules were placed into the aqueous phase. This led to a total of 128 POPE lipids, 64 geranate molecules, 64 geranic acid molecules, 64 cholinium molecules, and 7,808 water molecules in the system, resulting in a total of 44,288 atoms. The assembled system was briefly minimized to reduce any atomic clashes as the result of system assembly with Packmol. The minimization consisted of 5,000 steps of steepest descent followed by 5,000 steps of conjugate gradient. The system was then heated in the NVT ensemble from 0 to 100 K over 5 ps with 10 kcal/mol/ Å^2^ restraints applied to the lipid, geranate, and geranic acid molecules and ions. Subsequent to NVT heating, NPT heating was used to bring the system to a temperature of 310 K over 100 ps, and then to hold the temperature at 310 K over an additional 100 ps. No restraints were applied during the NPT heating phase of the simulation. The production phase of the simulation included 130 ns of completely unrestrained NPT simulation at a temperature of 310 K with anisotropic pressure scaling.

## Supporting information

S1 Text Supporting information table of contents and general methods used in this study. (DOCX)

S1 Fig ^1^H NMR spectrum of CAGE in CDCl_3_. NMR spectra were recorded on an Oxford 500 MHz spectrophotometer and processed via MNova 12. ^1^H NMR chemical shifts are reported in units of parts per million (ppm), relative to internal references for TMS (δ = 0.00 ppm) and residual CHCl_3_ (δ = 7.27 ppm).
(PDF)

S2 Fig ^13^C NMR spectrum of CAGE in CDCl_3_. NMR spectra were recorded on an Oxford 500 MHz spectrophotometer and processed via MNova 12. ^13^C NMR chemical shifts are reported in units of parts per million (ppm).
(PDF)

S3 Fig Conductance vs time for a representative CAGE preparation. Twenty mL of CAGE was freshly synthesized and conductivity immediately measured using a Hach HQ40d multimeter outfitted with an IntelliCAL CD401 conductivity probe. CAGE was then flushed with nitrogen, capped, sealed with parafilm and stored at room temperature in the absence of dessicants. Conductivity measurements were acquired over the course of six months.
(PDF)

S4 Fig Influence of 50% Ethanol Wash on MSSA Biofilm Viability and Sensitivity to Bleach. Biofilms of MSSA were cultured in CAMHB on MBEC biofilm inoculators as per the manufacturer’s instructions for 24 (left group) or 72 hours (center group). All biofilms were rinsed by immersion of the lid of the inoculator to remove planktonic cells, and were then immediately disrupted and enumerated (left plot in each group) or subject to immersion in 50% aqueous ethanol (3 X 10 sec), rinsed with fresh CAMHB, and then disrupted/enumerated (right plot in each group). MSSA biofilms (24 hr) were also treated with 10% bleach for 30 seconds (right group), followed by direct enumeration (left plot) or enumeration following an ethanol wash (right plot) as described above. Biofilms on the inoculator lid were disrupted from the surface via sonication (VWR Aquasonic P250D, power 4, 10 minutes) into a new 96-well plate filled with fresh CAMHB (200 **μ**L per well). Densities were calculated after dilution and enumeration on solid medium and values represent a minimum of three experimental replicates on each of three biological replicates (n = 9). The effect of the wash step observed here is consistent with that observed for biofilms of other strains examined in this report.
(PDF)

S5 Fig Average biofilm densities for examined strains. Biofilms of all strains were cultured in CAMHB on MBEC biofilm inoculators as per the manufacturer’s instructions for A) 24 or B) 72 hours. Biofilms were rinsed by immersion of the lid of the inoculator to remove planktonic cells, and the lid was then transferred to a new 96-well plate filled with fresh CAMHB (200 **μ**L per well) and were then dispersed with sonication (VWR Aquasonic P250D, power 4, 10 minutes). Densities were calculated after dilution and enumeration on solid medium and values represent a minimum of three experimental replicates on each of three biological replicates (n = 9).
(PDF)
