# Supplementary material for: Scope and efficacy of the broad-spectrum topical antiseptic choline geranate
Source: PLoS One. 2019 Sep 17;14(9):e0222211. doi: 10.1371/journal.pone.0222211 (PMC6748422; doi:10.1371/journal.pone.0222211)
Supplement: S1 Text — (DOCX) [file pone.0222211.s001.docx]

**Supplementary Information**

**Scope and Efficacy of the Broad-Spectrum Topical Antiseptic Choline Geranate**

**Short Title: Scope and Efficacy of Choline Geranate**

*Joshua R. Greene,^1^*^†^ *Kahla L. Merrett,^1^*^†^ *Alexanndra J. Heyert,^1^ Lucas F. Simmons,^1^ Camille M. Migliori,^2^ Kristen C. Vogt,^3^ Rebeca S. Castro,^3^ Paul. D. Phillips,^1^ Joseph L. Baker,^3^ Gerrick E. Lindberg,^1^ David T. Fox,^4^ Rico E. Del Sesto,^2*^ and Andrew T. Koppisch^1*^*

*^1^* Department of Chemistry, Northern Arizona University, Flagstaff, AZ, 86011, United States

^2^ Department of Chemistry, Dixie State University, St. George, UT, 84765, United States

^3^ Department of Chemistry, The College of New Jersey, Ewing, NJ, 08628, United States

^4^ Bioscience Division, Los Alamos National Laboratory, Los Alamos, NM, 87545, United States

^†^J.R.G. and K.L.M. contributed equally to this report.

|  |
| --- |

^*^ Please address correspondence to A.T. Koppisch (Tel 928-523-8893; FAX: 928-523-8111; email: [Andy.Koppisch@nau.edu](mailto:Andy.Koppisch@nau.edu)) or R.E. Del Sesto (Tel 435-879-7767; email: [delsesto@dixie.edu](mailto:delsesto@dixie.edu))

***Table of Contents***

**Page**

General Methods………………………………………………………………………….. 2

**Figure S1:** ^1^H NMR Characterization of CAGE.………………………………………… 3

**Figure S2:** ^13^C NMR Characterization of CAGE………….……………………………… 4

**Figure S3:** Variation in Observed Conductance over time in CAGE preparations.……… 5

**Figure S4:** Influence of 50% Ethanol Wash on MSSA Biofilm Viability and Sensitivity to

Bleach**.**………………………………………………………………………… 6

**Figure S5:** Average biofilm densities per mL for biofilms of examined strains…………. 7**General methods**

All chemical reagents were obtained from standard commercial suppliers. All antibiofilm assays were conducted on biofilm inoculators (Innovotech) following the manufacturer’s recommendations with minor modifications.  Antimicrobial data was collected on a BioTek Synergy H6 plate reader to measure absorbance at 600 nm. Clinical isolates of all pathogens were from an in-house strain library maintained at NAU. All microbial manipulation of pathogenic bacteria was conducted in a certified biosafety level 2 laboratory while following all associated safety protocols. The term MBEC in this manuscript refers to the minimal concentration of compound required for >95% inhibition of bacterial growth relative to growth of untreated controls. All MBEC assays were performed on a minimum of three independent biological replicates, each of which contained four experimental replicates per test.  MBEC values represent the highest determined from three biological replicates in cases where MBECs varied by 2-fold.
