# Supplementary figures and images for: Scope and efficacy of the broad-spectrum topical antiseptic choline geranate
Source: PLoS One. 2019 Sep 17;14(9):e0222211. doi: 10.1371/journal.pone.0222211 (PMC6748422; doi:10.1371/journal.pone.0222211)

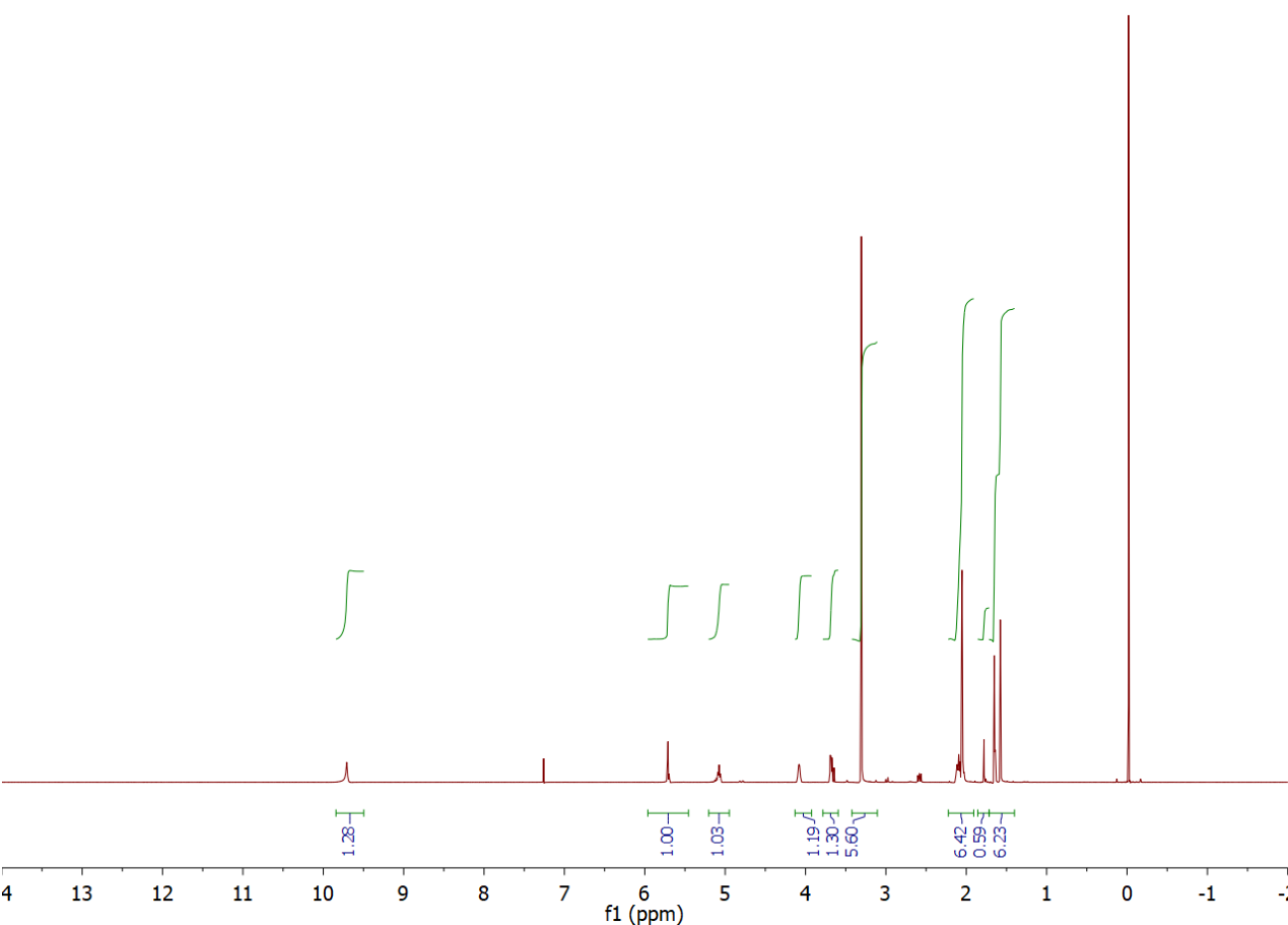

Supplement: S1 Fig — NMR spectra were recorded on an Oxford 500 MHz spectrophotometer and processed via MNova 12. 1H NMR chemical shifts are reported in units of parts per million (ppm), relative to internal references for TMS (δ = 0.00 ppm) and residual CHCl3 (δ = 7.27 ppm). (PDF) [file pone.0222211.s002.pdf]

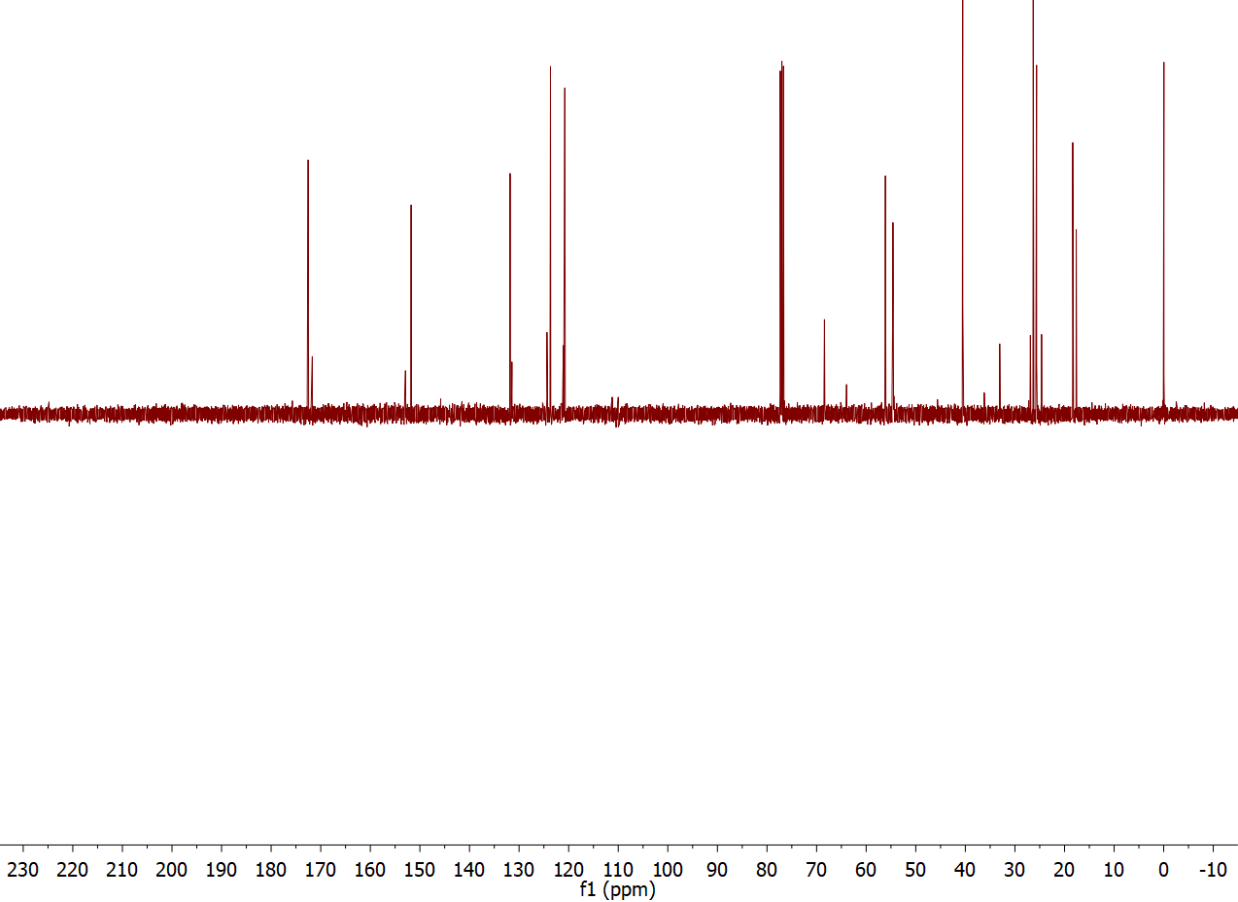

Supplement: S2 Fig — NMR spectra were recorded on an Oxford 500 MHz spectrophotometer and processed via MNova 12. 13C NMR chemical shifts are reported in units of parts per million (ppm). (PDF) [file pone.0222211.s003.pdf]

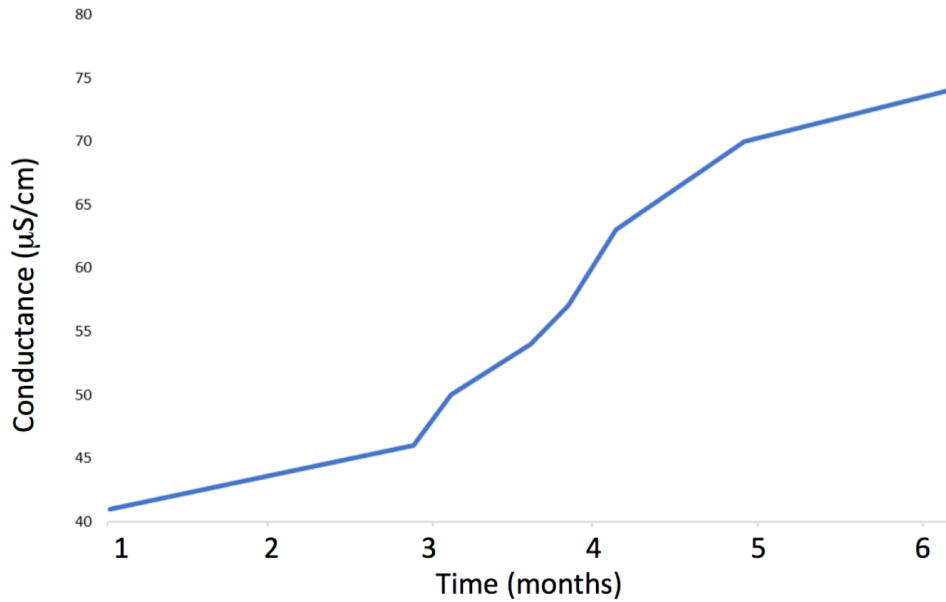

Supplement: S3 Fig — Twenty mL of CAGE was freshly synthesized and conductivity immediately measured using a Hach HQ40d multimeter outfitted with an IntelliCAL CD401 conductivity probe. CAGE was then flushed with nitrogen, capped, sealed with parafilm and stored at room temperature in the absence of dessicants. Conductivity measurements were acquired over the course of six months. (PDF) [file pone.0222211.s004.pdf]

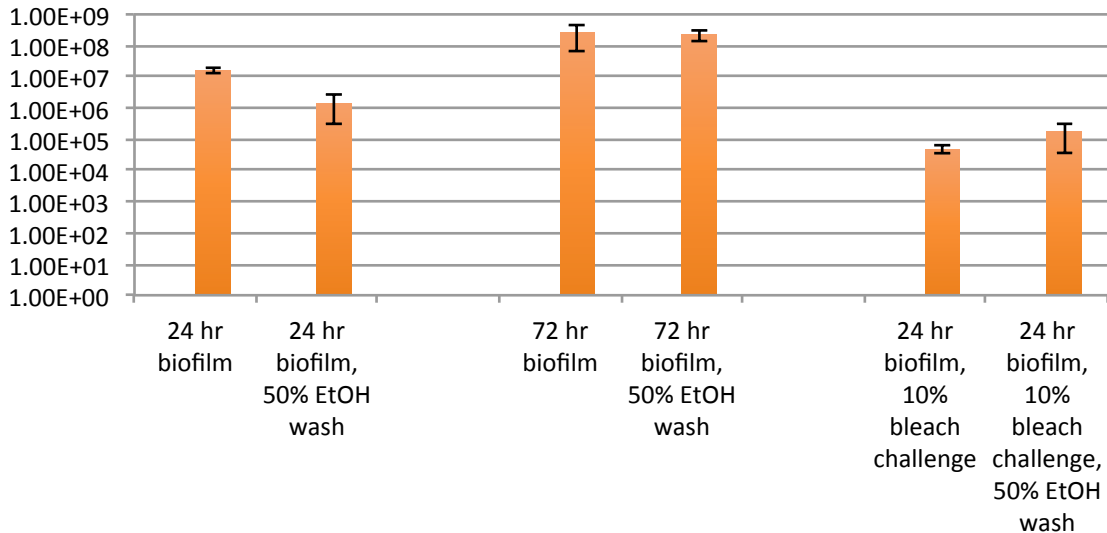

Supplement: S4 Fig — Biofilms of MSSA were cultured in CAMHB on MBEC biofilm inoculators as per the manufacturer’s instructions for 24 (left group) or 72 hours (center group). All biofilms were rinsed by immersion of the lid of the inoculator to remove planktonic cells, and were then immediately disrupted and enumerated (left plot in each group) or subject to immersion in 50% aqueous ethanol (3 X 10 sec), rinsed with fresh CAMHB, and then disrupted/enumerated (right plot in each group). MSSA biofilms (24 hr) were also treated with 10% bleach for 30 seconds (right group), followed by direct enumeration (left plot) or enumeration following an ethanol wash (right plot) as described above. Biofilms on the inoculator lid were disrupted from the surface via sonication (VWR Aquasonic P250D, power 4, 10 minutes) into a new 96-well plate filled with fresh CAMHB (200 μL per well). Densities were calculated after dilution and enumeration on solid medium and values represent a minimum of three experimental replicates on each of three biological replicates (n = 9). The effect of the wash step observed here is consistent with that observed for biofilms of other strains examined in this report. (PDF) [file pone.0222211.s005.pdf]

**A**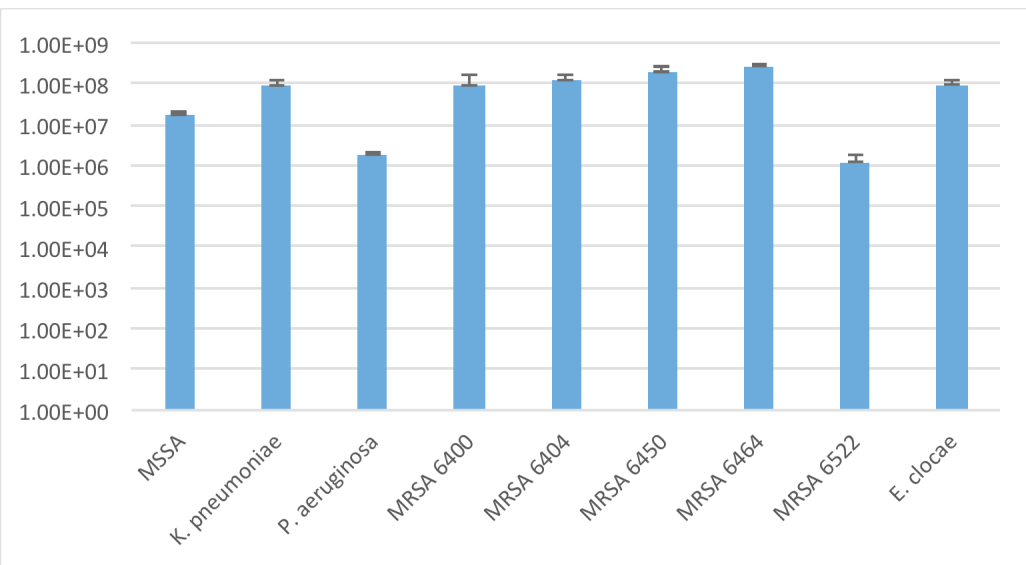**B**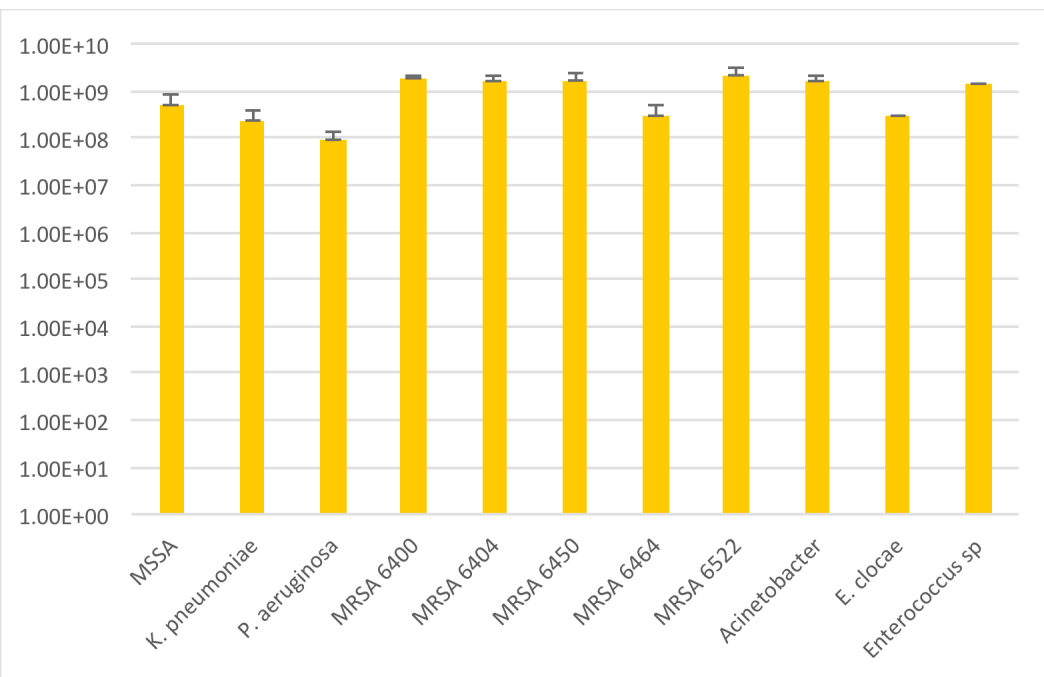

Supplement: S5 Fig — Biofilms of all strains were cultured in CAMHB on MBEC biofilm inoculators as per the manufacturer’s instructions for A) 24 or B) 72 hours. Biofilms were rinsed by immersion of the lid of the inoculator to remove planktonic cells, and the lid was then transferred to a new 96-well plate filled with fresh CAMHB (200 μL per well) and were then dispersed with sonication (VWR Aquasonic P250D, power 4, 10 minutes). Densities were calculated after dilution and enumeration on solid medium and values represent a minimum of three experimental replicates on each of three biological replicates (n = 9). (PDF) [file pone.0222211.s006.pdf]
